# Other Statistical Lives

**DOI:** 10.3390/ijerph181910369

**Published:** 2021-10-01

**Authors:** Max A. Greenberg

**Affiliations:** Department of Sociology, Boston University, Boston, MA 02215, USA; maxgreen@bu.edu

**Keywords:** risk factors, violence prevention, social equity, structural determinants, social environment

## Abstract

While recent scholarship has considered how algorithmic risk assessment is both shaped by and impacts social inequity, public health has not adequately considered the ways that statistical risk functions in the social world. Drawing on ethnographic and interview data collected in interpersonal violence prevention programs, this manuscript theorizes three “other lives” of statistically produced risk factors: the past lives of risk factors as quantifiable lived experience, the professional lives of risk as a practical vocabulary shaping social interactions, and the missing lives of risk as a meaningful social category for those marked as at risk. The manuscript considers how understanding these other lives of statistical risk can help public health scholars better understand barriers to social equity.

## 1. Introduction

In 2013, Harvey Fineberg, then head of the Institute of Medicine, raised a concern in *The Journal of the American Medical Association* that the “statistical lives” produced by public health data were far removed from the texture and meaning of “personal stories” that animate experience [1]. The statistical lives Fineberg describes and the mechanism of their production have recently been the subject of an appraisal by scholars focused on “algorithmic fairness,” who show how the tools of statistical analysis can reproduce the biases of the people and institutions who produce the tools [2,3,4]. Interrogations of algorithmic bias are vital, but insufficient to a consideration of social equity as it relates to the risk factors produced by public health research. This article, which draws on previously published data and revises earlier analysis [5,6], extends thinking from the social study of science and technology to consider the shifting and novel meanings of statistical risks outside of the formal articulations of research and policy. That is to say: statistics have lives of their own.

In the last 30 years, advances in computing technology and access to growing troves of data have multiplied the scope and power of statistical analysis and given rise to a staggering catalog of risks and protective factors. C. Wright Mills, in a tome of modern sociology, once argued that to understand any one person, we must situate their biography within the tides of history [7]. Risk data were not what he had in mind, but it has put his call into action to an unprecedented degree and into a novel form. Each individual can be understood as a cluster of data points in a stream of billions, tying past events to future possibilities. As it strips away the static of social life, risk analysis allows us to see how some kinds of events tend to follow others. The experiences of some become a prophecy for others. In this way, one person’s experiences with violence and trauma can cause someone whom they have never met to be labeled as at risk. This creates a kind of solidarity that borders on poetic: a stranger’s pain predicts my own and my future is bound up in their past. Like any other technology, statistical risk analysis is full of possibility: it opens up varied new ways to interact with one another and organize our social world.

Risk, Ian Hacking tells us, is a recent invention [8]. The first iteration of risk, which arose with simple probabilities, enabled the threats of the future to be met with insurance. Risk has since expanded to characterize a range of technologies of government; what Nikolas Rose described as “a family of ways of thinking and acting that involve calculations about probable futures in the present followed by interventions into the present in order to control that potential future” [9]. Peter Kelly, among others, worried that risk analysis could be used to justify an unprecedented level of “surveillance and intervention” into the lives of young people [10,11]. Writing in 1991, French social theorist Robert Castel argued that risk analysis had political and even existential consequences in the mental health fields. Castel wrote that prevention strategies “dissolve the notion of a subject or a concrete individual, and put in its place a combination of factors, the factors of risk” [12]. Castel contended that by looking for risk factors, professionals might find it difficult, if not impossible, to see the person before their eyes.

For over three years I observed interpersonal violence prevention programs in Los Angeles, talking to the facilitators who ran them, the managers that organized them and the young people who experience them. I set out to understand how the promise of prevention—to stop violence before it happens—looked in the lives of vulnerable young people. During the course of my research and in the years since, I became interested in the gaps between the way risk was described in the public health literature and how I saw it play out in practice. It was not the distant and calculating decision engine described in public health, nor was it the controlling force mapped out in the literature on power and governmentality. In my previous work drawing on these data, I provided some initial and under theorized analysis of statistical risk. Since then, I have come to see that risk analysis is best conceptualized and examined as a technology—akin to artificial intelligence, GPS or DNA tests—that makes possible new organizations of social life and new subjective experience as it reverberates through identities, narratives, and forms of social connection. Alondra Nelson, writing about the ways that the technological possibilities of DNA testing took on distinct social and economic meanings, described the “social life” of DNA [13]. Like DNA, risk factor analysis is a technology that tells us something about the past, which is also often is used to predict the future. Drawing on Nelson and other scholars working in the area of science and technology studies, it is possible to think expansively about what risk factors do and how they could be better used to counter social inequity. This article uses Nelson’s metaphor of lives to consider three questions about risk factors: How is lived experience quantified into risk factors? How are risk factors used in daily life by the professionals tasked with intervening upon them? What do risk factors mean in the life of the targets of their intervention?

## 2. Methods

This examination draws on data collected from a larger study of short-term interpersonal violence prevention programs implemented by a non-profit agency in Los Angeles from late-2009 through mid-2013. Programs were facilitated by a full-time staff of eight, as well as dozens of volunteers, and reached over 3000 young people across the city, predominantly high school students, each year. In all, I conducted participant observation at 20 public schools across Los Angeles county. All but two of the schools were selected because city, state, or federal agencies or private funders had designated the specific geographic community or demographic population as at risk of experiencing or perpetrating violence. The demographic makeup of the audience for the programs I observed—in line with the population of LAUSD—was almost entirely young people of color. The two school sites that were not marked as at risk were majority white schools in middle- and upper-income areas of Los Angeles county. The sessions that I observed lasted from forty minutes to over one hour and were often implemented to targeted groups of students in one session each week over multiple weeks. Some of the programs were intensive campus-wide initiatives that lasted between one day and a full week.

Like other agencies that implement youth programming, this one brought facilitators into schools to lead short lessons. It was in and around these lessons that my participant observation data were collected. Alongside 25 other volunteers, I went through the 50 h “violence prevention specialist” training and at times I facilitated portions of programs myself. While the level of activity of the volunteers varied, an email list with 60–80 names was used to fill volunteer opportunities in support capacities alongside paid staff. It was common for volunteers like me or other facilitators to observe program implementation. My time spent observing school-based programming varied considerably from week to week, since implementation depended on aligning timelines of demand, capacity, and grant funding. I might observe a single one-hour presentation one week and spend 36 h in the field during an intensive implementation to an entire school the next. During the course of my field- work, I did not record identifiable information regarding students, and I followed the guidance and standard practices of facilitators in any ethical matter that arose. In addition to my time in schools, I participated in organizational activities, such as trainings, program evaluations, and regular meetings of facilitators and other organizational staff, as well as informal meet ups totaling at least ten hours per month.

In school contexts, I arrived early to the classroom and stayed late to talk with teachers, administrators, and school personnel and to observe students outside the classroom. During presentations, I jotted real-time field notes in order to capture interactions, often in the margins of worksheets and curricula pages. On the occasions that I facilitated programming, I took advantage of regular breaks between classes to jot down detailed notes. I also dictated verbal field notes into an audio recorder immediately after leaving the field. I typed detailed field notes within 24 h and coded field notes for emerging themes. All names given are pseudonyms.

During the third year of fieldwork, I conducted 14 in-depth, semi-structured interviews with all of the facilitators employed at the organization during this time, as well as two volunteer facilitators. I also conducted in-depth, semi-structured interviews with 32 youth participants in violence prevention programming. Interviews ran between half an hour and two and a half hours and were conducted in break out rooms at the agency’s downtown office or in coffee shops at the participant’s request.

## 3. Other Statistical Lives

### 3.1. Past Lives

The past life of risk is experience. In order to make risk data, first the raw material of lived experience must become measurable through the data collection tools of risk analysis [14,15]. This process of quantification provides insight into large-scale patterns as it strips away noise and particularity to make patterns in causal links easier to draw. In this section, I recount the past lives of risk factors, before they solidified into data points, and show how the space between lived experience and the collection of risk data is a site of institutional meaning-making often influenced by racialized and gendered ways of seeing.

In order to take past events and making them numerically available, researchers collect and analyze data sets. This includes survey data that draw on the answers of representative sample of a population to questions about demographics and experience, such as having perpetrated or witnessed violence. Examples include the National Violence Against Women Survey or the National Intimate Partner and Sexual Violence Survey. Alternatively, bureaucratic records data from institutions such as police departments, emergency rooms, and rape crisis centers can show patterns of interactions with institutions, such as how many people called the police because of a violent partner or showed up at the emergency room with an injury. Survey data often show higher population-level rates of violence when compared with records data, which suggests hesitance in reporting to authorities. However, surveys too may be influenced by institutional meanings. This is especially noteworthy for instances of violence, as authority figures apply their own sense-making to events complicated by power, memory and emotion [16,17]. Institutions that serve the vulnerable and marginalized—both those tasked with punishment and support—are more likely to sort ambiguous events into more severe categories of action. The lines between drama and bullying, play fighting and violence, drug abuse and experimentation, sadness and trauma are often gray and decisions about what meaning to attach to events are influenced by institutional forces and perceptions of race and gender. This tendency toward the severe not only leads to disproportionately harsh punishments, but I theorize can also be solidified in formal record keeping data and by shaping the categories through which young people make sense of their actions on surveys. In this way institutional context, race and gender, among other social categories, powerfully shape the pathway between event and quantified data point by impacting the meaning applied to that event.

The attitudes and behaviors of the young people I talked to were a lot like their peers’ in middle-class and wealthy communities. Several used marijuana or drank recreationally. Most engaged in sexual activity. Nearly all had friends and loved ones involved in what would qualify as dating violence, although they rarely saw it that way. Those who had partners sometimes got into heated arguments. Several talked about situations in which they believed it would be okay to yell at or fight playfully with a partner. Yet, the meaning attached to these attitudes and behaviors when displayed by vulnerable young people was far more likely to be seen in more stark categorical terms.

Two of the most common risk factors that arose in my interviews with youth were showing signs of trauma and displaying aggression [18]. Every young person I talked to described one or both of these factors, yet I found that each risk factor encompassed diverse experiences and often obscured the process whereby something became recognized as trauma and violence. The stories that follow provide a glimpse of the underlying harms wrought by pervasive violence, trauma, and policing; and at the same time illustrate how data collection is more likely to capture forms of trauma and aggression that occur in institutional contexts occupied by vulnerable people.

Most of the young people I spoke with at one point or another “Display Aggression toward Peers or Display Other Aggressive Behaviors.” Aggression, after all, is a broad and often subjective marker. Young people often teased each other or play-fought, some threw a phone or tipped over a chair when they were angry or disappointed. Many played aggressive sports or violent video games. However, the use of these behaviors to mark individuals as aggressive often narrowed in on young men of color. For them, actions that might otherwise be seen as unfortunate accidents or as youthful roughhousing were more likely to be marked as violent, and, in turn, met with institutional responses.

When I interviewed Joe Moses, a young Black man, he was 15 years old and already six feet tall, although he bent his neck down, which made him seem smaller. He wore three strings of rosaries around his neck and his arms were dotted with burn marks and scars. We talked at a picnic table in the courtyard of his South Los Angeles high school. Joe was calm and shy, speaking with a slight lisp caused by a metal lip stud, while he told me about his experiences with police. He was arrested in eighth grade after a fight: “The dude was in class, he just kept bothering me, bothering me. So, he pushed me, and so, I socked him, and then I got arrested. They said battery assault, and then I was arrested.” Joe was arrested again after a teacher mistook play fighting for a real fight: “(The teacher) said if I didn’t go to the office, she was going to call the school police, so I said alright, I’m going to go to the office, and she still called for school police.” Even when his friend, the one he was play fighting with, told his parents that they were not actually fighting, they did not believe him. I asked Joe why he thought people seemed to worry that he was being violent, and he explained that it was because of his size: “Because I’m big, that’s why,” he said. The police cuffed Joe, put him into the police car, took him to the police station, booked him into the system, and then took him to juvenile hall. “It’s not a good place to be locked up in. Like, especially, if you’re not a violent person, you shouldn’t be there. It’s not a good place,” he told me. Joe’s experience mirrored the stories I heard from other young Black and Latino men, especially those who are big for their age, for whom what they saw as self-defense or horsing around was categorized as violence [19].

Crystal, a Latina young woman with multicolored hair, whose bag displayed a pin for a sexual assault awareness campaign told a very different story about a fight she had in eighth grade. She explained that while in middle school she was “going through stuff and didn’t know how to handle it that well.” She had a “low temper” and “would, like, just let it go on people.” We talked in a counselor’s office at the back of the school office suite:

*“I had this one girl, she was talking to me and I was like we were fighting, but it was on top of—it was like it was the stairs, and then like I pushed her. I didn’t mean to push her, but she like fell down the stairs, and I got—I got like in big trouble because they like the police took me, and like I got a ticket for like fighting, and my mom was all like in my business, and everything. And then the—the girl, I mean, the girl was OK, but they still saw it as like I tried to harm her.”*

The police wrote Crystal a ticket, but her repercussions paled in comparison to Joe’s: “I had to go to court, but I just paid it off, like, with money, because I don’t want to, I don’t want to do community service, so I just paid it off.” Crystal’s light punishment, and her ability to get out of it by paying, was in part a byproduct of her gender, race, and class. Similar behaviors, such as play fighting, are often interpreted differently depending on the social location of the actors. These interpretations are then more likely to be translated into institutional data and self-described meanings.

Most of the young people I interviewed could readily be tagged with the risk factor “Depressed, Anxious, or Have Other Symptoms of Trauma.” Many had lost loved ones to illness or violence, had family members in jail, had witnessed shootings, or had been beaten up or harassed. While trauma is a fact of life for young people in every city and town, it was clear that young people in the marginalized neighborhoods of Los Angeles dealt with a disproportionate amount. Flora, a Latina student, told me that she believed that violence was everywhere she went “It’s not only South Central, it’s West LA, it’s in the mountains. Yeah, it’s everywhere. And like where I used to live, um, I grew up in another country and it’s like next door, in front of my house, right in my door. Like yeah, everywhere.”

I talked with Flora the day after a group of young people went to see Fruitvale Station, a film that chronicles the last day in the life of Oscar Grant, a Black man who was shot and killed by a white transportation officer in Oakland while lying on the ground with his hands above his head. When I asked Flora, who is Latina, about the movie, she explained that it reminded her of what happened to her father:

*“I felt like I was living (it) again, like oh my god, no, no. And um, just looking at the baby girl just, being with her father, just having fun and being a kid with a loving father. And I (said) like, (he’s) just another one. If my dad died, why not him? In like, in my opinion, my dad was a good person. And why him?”*

The death of Flora’s father was unquestionably traumatic. But through her participation with a local nonprofit and through long conversations with her mother, she had come to see it as a too common occurrence. Flora explained how, since Trayvon Martin had been killed by George Zimmerman, she had begun to shift the way her own trauma and loss shaped how she acted: “It just made me change so much about the way I think now. And my mom is like ‘Oh you know you’re perfect now’ and I’m like ‘No, I’m not perfect. I’m just trying to change.’” Flora had begun to advocate against violence and for social justice. Her trauma was not just a risk. Flora’s loss became a catalyst for her to work to change society and herself.

Scott had been largely home-schooled because his mother moved often in order to stay away from an abusive man. When his mom took a job outside of California Scott, eager to maintain the friendships he had built, stayed in Los Angeles in a small apartment with his best friend. In what seems to have been a tragic case of mistaken identity, Scott and his friend were shot leaving their apartment complex. His friend died and Scott was badly injured and needed major surgery. Concerned about losing her job, Scott’s mother drove back and forth to work in a neighboring state and the staff of a local agency cared for Scott when she was away.

The loss experienced by Flora and the violence enacted upon Scott are just two among the many traumatic events that happened to the young people I spoke with. And at the same time, their stories show the ways that support institutions have arisen in places with high rates of violence and trauma. Just as punitive institutions shaped the way young people made sense of aggression and violence, supportive organizations and adults could shape the meaning of trauma and provide some counterbalance against the forces that can bend trauma into violence. However, they did so by making clear that what had happened was trauma and deserving of formal support.

The past isn’t a fair place. It is shot through and torn up by systemic injustice. Although violence occurs across every corner of society, it is multiplied by structural racism, heteronormativity, environmental hazard, deprivation, and so on. Difficult pasts are unequally distributed, sorted along lines of marginalization and vulnerability and which live on in the form of trauma. At the same time, institutions and organizations in vulnerable communities are designed to make legible and intervene in these instances, far more than in wealthier communities with access to private forms of support. Risk analysis relies on data from organizations with institutional pressures to catalog harms as a way to show their efficacy. In this way, high rates of violence and trauma also produce institutions invested in marking events and experiences with categories that warrant intervention and in doing so influence not only the formal sorting processes, but the ways that individuals describe their own lives.

### 3.2. Professional Lives

Prevention is built upon an institutional apparatus that links research with frontline workers. For the practitioner of prevention, risk factors arrive in research briefs and PowerPoint slides, fully realized and imbued with institutionalized moral authority. Risk—its accounting and transformation—is the currency of their daily work and the source of their livelihood. To doubt its existence would be akin to a doctor doubting medicine or a stockbroker doubting money. Risk factors function as one part of a professional vocabulary that can be used to get things done: account for time spent, mark actions, sort groups and individuals, provide services for them, and justify abnormal action. In short, risk provides the rationale for all that they do. At the same time, this vocabulary distances practitioners from the real social and human aspects of their work.

Statistical data do not simply reflect reality, but, as scholars have argued, actively creates it. Statistical representations “invent and transform collectivities and social reality. These have effects on how people conceive of themselves and others” [20,21]. We can see this in action, as risk data take on a life of their own and reverberate across grants, curricula, fact sheets, and the formal and informal discourses of risk work. Technological inventions such as risk analysis are also cultural processes, producing value differences, redrawing symbolic boundaries, and constructing new kinds of subjects. In professional settings, risk analysis does not just recount the scatterplot of a life, it gives language to the stories that practitioners can tell about how lives unfold.

The accumulation of risk factors is what directs prevention programming to one population or another. From the 10,000-foot view, structural harm in the past becomes risk in the present. For practitioners, risk worked as a blanket justification for their intrusion into lives. Many practitioners said that every youth they worked with was at risk for one reason or another. Ella told me, “In the communities I’ve worked with, every single (youth) is high-risk just because of the environment that we live in.” I asked if she just meant high risk for violence, and she responded: “At risk for anything, drug use, alcohol, pregnancy. It’s overall just high- risk.” The high rates of risk lead to widespread application of a sort of fundamental at-riskness that is carried in bodies of color in particular places.

Janet Shim, writing about the social origins of heart disease, describes the hidden “politics of causation” in medicine. We often think that race is associated with heart disease, but Shim argues that it is structural racism—the way that people of color are treated—that causes increased rates. To make an argument about causality means picking a causal variable to start at and inevitably erasing some aspect of causation [22]. Drawing on risk data, prevention locates risk in the small-scale world of behavior and personal experience, disconnected from institutions and identity. The causes are made to be interpersonal, emotional, and habitual, but not economic, medical, or political.

This politics of causation was evident in the practitioners I studied in Los Angeles, who often described seeing “red flags” in young people. The category, its contents, meaning and implications were largely taken for granted. Red flags were everyday expressions of the risk factors described in public health research: some red flags referred to personality qualities or behaviors, while others were based on environmental attributes. The practitioner I met had learned to see risk in all parts of daily life. As one practitioner told me, “I wish I could turn it off when I’m listening to music or watching TV. I wish I could turn it off. Like in my house, like if I hear a woman scream. I wish I could turn it off when I’m talking to my friends because I’m sure they would appreciate that. This is just the way I look at things at this point.” Not long after beginning work as a program facilitator, I felt like I could see red flags too, flashing like alerts over the heads of the students as they ricocheted through the halls, or as they recounted their experiences in interviews.

I sat across form Paula as she ticked through her life history. Early in her life, she witnessed a shooting. She told me: “We were just walking down the street and there was a drive-by and then it was like right in front of our faces and my mom was super scared. She just threw us on the floor and she was like ‘Get down!’” *Red flag: witnessing violence*. This was one of many incidents of violence in Paula’s life. When I asked her where she saw violence, she said, “Well, like everywhere. I see it in a lot of places. Not just here in LA, also like the valley. I used to live in (the valley), you know, there was violence there. Violence is everywhere. It’s like you can’t escape it.” *Red flag: high violence environment*. Paula told me that she was a good student early on, but then things changed when her sister, who had been her “role model,” was arrested and sent to juvenile hall. *Red flag: few close connections*. Her brother was in a gang and she almost joined one too. *Red flags: gang affiliation*. She explained: “The people around me they weren’t good role models either and then, like my environment, my surroundings, like the park, it was filled with gangsters.” *Red flag*. In middle school, Paula fell under the influence of some “older kids” who, like many teenagers, wanted to “try everything.” She became “a big ditcher,” began experimenting with drugs, and was “not good in school.” *Red flags, again and again.*

Ann Ferguson [23] has argued that one way in which public school teachers and administrators criminalize young men of color is to adultify them, depriving them of their social position as children—and all the support for experimenting, learning and growing that it affords—and placing them in a framework of adult rules and regulations. In their professional application, risk factors enact a distinct and parallel process whereby practitioners treat young people not as individuals with specific pasts, but as potential futures to be managed. It is a distinct causal construction that erases the forces that produce the accumulation of events called risk in the lives of vulnerable young people, and constructs risk factors as embedded in specific bodies, with their behavior as the only medium through which to bend the causal flow. To begin the causal story with risk factors—trauma, disengagement, isolation, or distrust—erases the forces that gave rise and shape to them. These things are not just factors that produce harm, they are harm themselves, beget by specific arrangements of history and structure. Young people, often black, brown, queer, or poor, are the object of intervention today because bad things happened to them before now. Yet, this is lost in the vocabulary of professional risk.

Red flags are not the only way to make sense of the unfolding of a life like Paula’s. The other way to understand Paula, the way that she preferred to be seen, was as someone who took control of her own life in the face of failing systems and strained connections. Much of Paula’s worldview was shaped by broken institutions, and as a result she learned to be fiercely independent. The structure of prevention and the vocabulary of risk meant that most adults never saw this confident, determined Paula. Whether Paula’s story is framed as a tale of a young woman navigating broken systems or mounting risk factors not only determines how we make sense of the most important moments in her life, it sets the stage for the kinds of connections that can be formed between her and the practitioners there to change her future.

In this way, risk factor analysis has shaped the way those most immediately tasked with countering structural inequality experience it, converting it into landscape of individual trajectories, checked with obstacles to be rerouted around. Practitioners expressed deep commitment to the work of prevention and to the young people they met. Yet, they found it less fulfilling and meaningful because they were removed from the full humanity and social relations that shaped young people’s daily experience.

### 3.3. Missing Lives

By formal and informal measures commonly used in the professional violence prevention field, every young person I spent time with was “at risk.” Yet, regardless of which young people I talked to, I found that the narrative of risk that animated troves of research and millions of dollars of funding for prevention programs made little sense to the young people it was supposed to help. For example, consider this conversation from my interview with Hendrix:

Max: Have you ever heard the phrase “at risk”?

Hendrix: At risk? Yeah.

Max: Yeah, what does it mean? Where have you heard it?

Hendrix: I mean, I haven’t heard it a lot, but I know what it means.

Max: What does it mean?

Hendrix: It means, like, something that’s, like, you’re walking on egg-shells pretty much.

Max: Do you feel like you know people who are at risk?

Hendrix: At risk of—of just period?

Max: Of anything. Yeah.

Hendrix: Yeah.

Max: How about at risk of violence?

Hendrix: At risk of violence, no.

Max: No, okay.

Hendrix: I don’t know a lot of people. I mean, I know a lot of gang members. That’s, like, violence, right?

Hendrix was 16 years old when we talked in an empty classroom after school let out. The small “continuation” high school Hendrix attended was in a storefront on a major thoroughfare, in a predominantly Black and Latinx neighborhood in South Los Angeles. There were 80 total students enrolled, only 50 of whom regularly attended. The school had a reputation for accepting students who had struggled elsewhere, and many of the students were on probation, in foster care, or had been pushed out of other schools, which led some students to describe it as a “last- chance school.” I expected that of all the youth I talked to, the ones there would have picked up on the language of risk and red flags that swirled around them, but they had not. Most, instead, shared Hendrix’s confusion. This was also true of young people from across Los Angeles, many of whom were in traditional public schools and excelled at academic pursuits.

Scientific and technological advances produce ripples in the social worlds of those they impact. In her book about the social life of DNA, Alondra Nelson charts how the science of DNA was used to make claims on identity and to contest it, to seek profit and citizenship. Other scholars describe formulations of identity, collectivity, tension, rebellion and more that emerge from scientific constructs. However, there does not seem to be a social life of risk factors for those young people marked as at risk. They do not challenge the designation, resign themselves to it, or vie to retake its meaning in pursuit of agency. Few even recognize the term.

For Hendrix and other people who would fit the designation “high-risk” things that were statistically designated as risk factors felt commonplace. Angel, a 19-year-old Latino young man with deep-set eyes and a level voice, explained how he saw his life compared with other people his age: “I think it’s normal, the same thing (as) like every 19-year-old, like certain things we go through, like, uneducated, a lot of 19-year-olds are like that. Not being employed. These troubles are byproducts of the social and economic context in which Angel lives, but from his perspective they are simply how life is. As Angel explained, in underserved communities, risks are common and therefore young people develop a locally defined sense of the baseline level of risk. Angel: “I know everything about violence. Like that’s all I know. Like all I know is about violence. Like everywhere I’m surrounded by, it’s like violence everywhere.” He continued: “I feel like violence was just there for me.” Young people rarely described marginalization and violent events as risk factors, but instead as everyday facts of life. From their perspectives, living in their community, risk was everywhere and therefore in a way, nowhere.

Several times, when I asked youth where they saw unhealthy or violent behavior, the answer was simply “everywhere.” Gina, who at 25 was by far the oldest youth participant I interviewed, was part of a performance-based anti-violence program run out of a dance studio. Gina saw herself as a role model for other participants, some as much as a decade younger than her. We talked in a break room alongside the mirrored space where the group was practicing for an upcoming performance. Gina was one of the few who described aspects of her life using the vocabulary of risk. She said that “a lot of the warning signs that one would never think or one is so used to, such as, you know, fighting all the time for example, in my community are a normality. So to (learn) that that’s a warning sign for me was, well, I hear (warning signs) all the time.” She continued, “You hear ambulances, police, your typical city. So, there have been calls on domestic violence maybe two houses down from me. A child was abused in the house diagonal to mine. There was a juvenile delinquent who actually was a friend of my brother’s when we were young. He grew up with the wrong crowd.” Gina found it bizarre to make sense of everyday life in her community, among her neighbors, peers, and family, through the lens of risk. As Gina described, in underserved urban communities, risks are multiple and proximal but residents rarely identify them as such because they are attached to specific individuals and embedded in their local social world. It is hard to see your neighbors or your brother as embodiments of risk.

Like many young men marked at risk, Hendrix had been in trouble for most of his educational life. He was skeptical of schooling, but he did well enough to get by when he tried. At a young age, he was pulled into a pattern of suspension and expulsion, a pattern far more common among young men of color. He was suspended in second grade for “poppin’ a firework in class.” The harsh discipline at the school was disconnected from lessons at home. He explained, “My mom didn’t know. She thought that I was just being sent home for some reason. In school I’ve been suspended sixth grade, seventh grade, eighth grade, ninth grade, tenth grade been suspended. And I’ve been suspended, like, three times every year.” When I asked him what kinds of things he was suspended for, he explained:

*“I used to get in a lot of fights with people. I used to do a lot. And my principal didn’t like me. She hated me. She hated me so much. She used to try to find little things, she, I guess, I’m pretty sure she sent someone to write “Fuck Ms. Curtis” on the wall, right. And it was the teacher that I had just had a parent-teacher conference with. This principal hated me so much she brought me up to the office, she had a paper of mine—I don’t know how she got a paper of mine, right. She was like, “It’s the same writing that was on this wall,” tryin’ a suspend me. She was like here comes one administrator. But I was, I mean, I wasn’t stupid. I was like, OK, you can bring them all in, I didn’t do it. And do you have proof that I did it? Why are you accusing me that I did it? You know, she didn’t like me. And she knew that I was smart, and I wasn’t stupid, but that was always her motto, like, tryin’ to suspend me, tryin’ to take me out of school. So when she found out that I was leaving the school, she was like, “Yay! Thank God he’s leaving.””*

After Hendrix left that school, he went to a charter school in the valley, but he was only there for a short while before it was closed down, “’Cause my principal was corrupt. Corrupt principal.” Hendrix went to another school, but that didn’t last either: “I was a bad kid. I moved to (another school). After there—they didn’t want me there—I moved there for a month, and I went to a continuation school. I went there and then I came here. Yeah, so I’ve been at, like, five (high) schools.”

At no point in his story does anyone tell Hendrix he was at risk. Instead, he found himself navigating a landscape of relationships with authority figures marked by antagonism and misunderstanding. One principal hated him, another was corrupt, another thought of him as a “bad kid.” Without having data from these figures, it is difficult to be sure that those around Hendrix had heard of the term at risk, though given the widespread dissemination of the term, it is likely that they did.

In the context of violence prevention programs, I saw practitioners draw on a robust language and organizational apparatus dedicated to finding, marking and transforming risk factors and yet, I never saw them explain that logic, even when young people explicitly asked why they were there. Instead, practitioners described themselves as someone there to help students make healthy choices, without suggesting why their choices were otherwise suspect. Given the apparatus of health communication in public health, it is curious that the vocabulary of risk is not at the heart of contemporary public health efforts with youth. In the most generous rendering, it promises to detach stigma and posit young people not as bad or good, but as bundles of unrealized potentialities.

I talked with Angel in an empty classroom at the small charter school he attended in Los Angeles. On the walls around us were students’ “dream collages” for a school assignment, pictures cut out of magazines and pasted onto construction paper of what they wanted for their future. One was covered in expensive cars and a swimming pool, another with models in swimsuits, chopped out of their surroundings. As a society we tend to talk about young people as all future and no past, endless options unspooling ahead of them. Angel, like many young people I talked to could not get away from the past. It was not just his own violence that dogged Angel, but his home and community life also bore scars. He told me that there was “all this violence and I knew I was at that place, like I was doing violence, violence was going on in my life.” It used to be that every time he got angry, it would “come down to violence” because he did not care about consequences at that moment. He would punch walls. One time, he punched glass and badly lacerated his hand. All of that accumulated violence meant that Angel was at risk. It was this fact that had brought me into his classroom. Angel explained that even before the program came into his classroom, the way he thought about violence was changing “after a couple experiences and, like, getting my things together and moving out, and getting my head out the streets.” He had started to gain control of his temper. “It’s changing little by little,” he told me. Still, his history would not let go easily. Angel told me that “certain mistakes from the past” meant that he had to be careful in his neighborhood. The past had its own inertia beyond Angel’s control. Even as he tried to change his life, the past pulled at him. It was not just his past, after all; it lived on in the stories of others, in the geography of his neighborhood, in flashes of memory.

I found that chances to work through complex and compounding histories, were rare in the lives of the young people and many eagerly turned our interviews into a chance to talk about their pasts. The driving logic of risk data is that the past matters and yet practitioners, whether for occupational or interpersonal reasons, acted as if the past—the thing that brought them into the lives of young people—was inaccessible. This meant that Angel had never heard of post-traumatic stress disorder, nor did he know that his experience of violence was not rare, nor did he get credit for his efforts to change and support for navigating a treacherous terrain. The push for “trauma-informed” interventions approaches this concern, but does so from an angle. Rather than open up the past, and the harms associated with it, it places the past in box separate from risk. Trauma-informed practices, at least as I observed them, largely took the form of teaching practitioners to bracket, avoid, and outsource conversations about past harm. In doing so, they further distanced statistical risk from lived experience, sorting emotions and behaviors into separate silos of institutional expertise.

## 4. Conclusions

In this manuscript, I have drawn on science and technology studies to consider the “other lives” of the risk factors articulated in public health research as they emerged in my previous research into interpersonal violence prevention programs. This focus on other lives provides a window out to the ways that public health research reverberates through the social world. In doing so, it opens up novel ways to think about social equity and new avenues for research.

Before public health data are collected, its raw materials exist as experiences. The meanings attached to these experiences, and thus the likelihood that they are inputted into particular categories during data collection and analysis, depends on the way that authority figures and experts co-construct them with the people who lived through them. Attention to what I have called the past lives of risk factors highlights racialized social processes of perception and authority that underlie data collection and quantification. Armed with existing definitions of risk factors and the questions and processes used to elicit them, researchers should enter into a diverse array of contexts and use interviews and observations to examine gaps between existing conceptions of risk factors and the ways they are constructed in practice. For example, is yelling at a teacher equally likely to be seen as a sign of aggression in a mostly Black school and a mostly white school? How does institutional context matter for the way that some events become seen as traumatic?

As other public health scholars have argued, risk factors can individualize risks, lodging them in particular bodies, and draw attention away from structural forces [24]. A structural risk approach may sensitize risk researchers to enduring and systemic forces of inequality, but it is not clear what it would do for practitioners, who regularly interact with those marked as at risk. The causal construction of risk, regardless of what it is, shapes the ways that practitioners make sense of their work. A causal metaphor that sees risk flowing from outside structures and into bodies, whether the individual or structural is privileged, saps agency from those marked as at risk and positions the practitioners at a distance. Further research should investigate the levels of work satisfaction and burnout in prevention practitioners and consider how more robust connections with those marked as at risk may be of benefit. It should also consider new metaphors for the way that risk works. For example, we could conceptualize risk engines as things that produce risks and work alongside community members to challenge and dismantle them.

Despite the central place of risk in the research, organization, and implementation of prevention, practitioners avoid telling young people that they have been marked as at risk. This is surprising given that in interviews most practitioners describe risk as a legitimate assessment of the young people they work with and as a technology that captures structural and systemic inequalities, not personal failings. However, the language of risk could open up uncomfortable interactional dynamics, particularly around the question of what made someone at risk. The answer to that question, as I have shown, is either their demographic characteristics, or something in their past, both of which practitioners were ill equipped to explain or justify. Public health needs to grapple with what it would mean to be clear with the objects of prevention about how risk factors are constructed, applied and consequential. Without doing so, prevention programs can be little more than a temporary stranger passing through the lives of young people. Behaviors are shaped in part by the stories we tell to make sense of the churn of experience. What would a young person say about themselves if they knew they had been marked as at risk? Maybe they would resist the description, but in doing so they may find new ways to think about themselves and those around them. What would a young person do if they had access to the technology of risk analysis? What kinds of new identities, connections and ways of being might bloom?

## Data Availability

Due to privacy concerns, the data (fieldnotes and interview transcripts) are not available.
